# Evaluation of reference genes and characterization of the *MYBs* in xylem radial change of Chinese fir stem

**DOI:** 10.1038/s41598-021-04406-1

**Published:** 2022-01-07

**Authors:** Kui-Peng Li, Wei Li, Gui-Yun Tao, Kai-Yong Huang

**Affiliations:** 1 Guangxi Key Laboratory of Superior Timber Trees Resource Cultivation and Key Laboratory of Central South Fast-Growing Timber Cultivation of Forestry Ministry of China, Guangxi Forestry Research Institute, Nanning, 530002 People’s Republic of China; 2 College of Landscape Architecture and Forestry, Qingdao Agricultural University, Qingdao, 266109 People’s Republic of China; 3 Research Institute of Forestry, Chinese Academy of Forestry, Beijing, 100091 People’s Republic of China

**Keywords:** Transcription, Plant development

## Abstract

The radial change (RC) of tree stem is the process of heartwood formation involved in complex molecular mechanism. Chinese fir (*Cunninghamia lanceolata* (Lamb.) Hook.), an evergreen species, is an important fast-growing timber tree in southern China. In this study, the top four stable genes (*IDH*, *UBC2*, *RCA* and *H2B*) were selected in RC tissues of 15 years old Chinese fir stem (RC15) and the genes (*H2B*, *18S*, *TIP41* and *GAPDH*) were selected in RC tissues of 30 years old Chinese fir stem (RC30). The stability of the reference genes is higher in RC30 than in RC15. Sixty-one *MYB* transcripts were obtained on the PacBio Sequel platform from woody tissues of one 30 years old Chinese fir stem. Based on the number of MYB DNA-binding domain and phylogenetic relationships, the *ClMYB* transcripts contained 21 transcripts of MYB-related proteins (1R-MYB), 39 transcripts of R2R3-MYB proteins (2R-MYB), one transcript of R1R2R3-MYB protein (3R-MYB) belonged to 18 function-annotated clades and two function-unknown clades. In RC woody tissues of 30 years old Chinese fir stem, *ClMYB22* was the transcript with the greatest fold change detected by both RNA-seq and qRT-PCR. Reference genes selected in this study will be helpful for further verification of transcript abundance patterns during the heartwood formation of Chinese fir.

## Introduction

Gymnosperms and angiosperms are categorized into woody plants and herbaceous plants. The stem of woody plants is mainly composed of secondary xylem^1^. Wood formation in tree species is performed with the complex coordination of cell differentiation and secondary cell wall (SCW) thickening, which are two highly ordered processes initiated from the vascular cambium^2^. The structure of gymnosperms wood is relatively simple, consisting of tracheids, rays and parenchyma. On the other hand, angiosperm wood is structurally more complex, composed of vessels, fibers, rays, and parenchyma^3^.

The vascular bundle contains phloem cells, cambium cells, and xylem cells from the outer layer to the inner layer of tree stem^4^. Wood developed from several major steps is mainly composed by secondary xylem cells of trees^5^. Sapwood (SW) is derived from vascular cambium has living cells and is located in the outer layers of wood. Heartwood (HW) formation is the process of sapwood senescence with the death of parenchyma cells and the cease of all physiological activity. The transition zone (TZ) is located in between the SW and HW^6^. Parenchyma tissue contained ray and axial parenchyma cells in secondary xylem represents the bulk of living cells. Ray parenchyma cells are involved in HW formation. Programmed cell death (PCD) plays an important role in secondary xylem cells and HW formation in woody plants^4^.

Gene expression level analysis plays an essential role in molecular biology^7^. Real-time quantitative polymerase chain reaction (qRT-PCR) is a widely applied technique for precise quantification of gene expression and is widely used in modern biology due to its prominent advantages of high sensitivity, specificity, and repeatability^8,9^. Stable internal reference genes are essential to normalize gene expression for obtaining accurate measurement results in the qRT-PCR assay^7^. Reference genes are usually derived from housekeeping genes, which maintain the basic processes of cell cycle^10,11^. However, the housekeeping genes expression levels vary greatly in species, tissues, and under certain experimental conditions^12^. Therefore, selecting stable reference genes according to the different sample types and various experimental conditions is the primary step for a reliable gene expression analysis by qRT-PCR assay^13^.

Transcription factors (TFs) are proteins capable of controlling the expression of the target genes and play an important role in regulating of multiple biological processes. According to conserved domains, TFs can be divided into different gene families. The MYB family members contain a conserved MYB binding domain consisting of one to four imperfect tandem repeats (R) of about 52 amino acids at the N-terminus. Depending on the number of the adjacent MYB repeats, MYB genes can be classified into four major classes: MYB-related (1R-MYB), R2R3-MYB (2R-MYB), R1R2R3-MYB (3R-MYB), and 4R-like MYB (4RMYB)^14^. MYB proteins, especially R2R3-MYB proteins, are the key factors regulating plant development, primary and secondary metabolism, responses to biotic and abiotic stresses, lignin biosynthesis, xylem secondary cell wall (SCW) formation, and PCD^15,16^.

The radial change (RC) of tree stem is the process of heartwood formation and the process involved in PCD of living cells including ray and axial parenchyma cells. Up to our knowledge, there were few previous studies related to the selection of suitable references genes for target gene expression in RC of tree species stem. Chinese fir (*Cunninghamia lanceolata* (Lamb.) Hook) is a fast-growing native, allogamous, long-lived tree species mainly distributed in southern China and has been planted for timber production for over 1000 years^17^. Chinese fir accounts for 20–30% of the total commercial timber production in China^18^. The tree species is a gymnosperm in the cypress family Taxodiaceae. Taxodiaceae traditionally defined includes 10 genera and 16 species^19^. The somatic chromosome number of Chinese fir is 2 *n* = 2 x = 22 which is in concurrence with other diploid members of Taxodiaceae^20^. The diploid genome sizes of Chinese fir (28.34 pg/2C) is the largest among Taxodiaceae^21^.

In the present study, expression stability of 12 candidate reference genes were assessed by qRT-PCR in RC of Chinese fir stem. Phylogenetic tree was constructed to predict the biological functions of *ClMYB* transcripts. The expression profile of 61 ClMYB transcripts in the RC were analyzed by RNA-seq. Furthermore, the expression patterns of 25 *ClMYB* transcripts were validated by qRT-PCR using the four selected reference genes for normalization. This research provides a new understanding of suitable references genes in RC of tree species and expression pattern and biological functions of *ClMYB* transcripts.

## Results

### Expression stability of candidate reference genes

The primers of 12 candidate reference genes were listed in Table 1. The specificity of each primer pair was verified by 2.0% agarose gel electrophoresis (Fig. S1) and melting curves analysis (Fig. S2). The primer pair with only a single qRT-PCR product was selected. Amplification efficiency and R^2^ were counted by the standard curves (Fig. S3). BestKeeper ranks the candidate reference genes according to the Pearson correlation coefficient (r). The reference gene with high stability has a higher r value. In the radial change of 15 years old Chinese fir stem (RC15), *IDH*, *RCA, UBC2*, and *RPL2* ranked as the top four stable reference genes (Table 2). In radial change of 30 years old Chinese fir stem (RC30), the results indicated that the *18S*, *H2B*, *IDH*, and *GAPDH* were the top four stable genes (Table 3).

**Table 1:** Primer characteristics of 12 candidate reference genes for qRT-PCR.

Gene symbol	Gene description	Primer sequence (forward/reverse)	Product (bp)	Amplification efficiency (%)	R^2^
*GAPDH*	Glyceraldehyde-3-phosphate dehydrogenase	GGTCACTGGTTCTGCCAAAT/TGACAACGAGTGGGGATACA	101	88.1	0.999
*H2B*	Histone H2B	TATCGGAATTTCCAGCAAGG/ATACCTGGCCAATCTGGATG	97	92.0	0.999
*TIP41*	Transcription and replication	CAAGCCAGGTCTCTCCAAAG/GAGAGCAGGACATGGAGGAG	199	94.1	0.994
*18S*	18S ribosomal protein	GCTTCTTGCTCTACCGGATG/AATGCAACATCAAGCATGGA	144	90.5	0.998
*IDH*	Isocitrate dehydrogenase	CTTTTCATGCAGTCCCAGGT/TTGCGCTAGCTGAAGCTGTA	122	87.7	0.999
*PXMP2*	Peroxisomal membrane protein	TAGTGCAGGCTTGAGGCTTT/AGTTTCCAGTTTGCCACCAC	159	101.4	0.999
*RCA*	Rubisco activase	GCTTGCCAATGCTCTCTACC/TTTTATTGGGCTCCAACCAG	208	102.7	0.996
*UBC2*	Ubiquitin-conjugating enzyme	TTGTTTTGGCAGTCTGCTTG/GCTCGTTTCTGATGGCTTTC	216	84.6	0.998
*RPL2*	Large subunit ribosomal protein	CCGCTGCTCTTTATCCTCAG/GCATCCGAGAGGGATTATGA	223	100.9	0.999
*DnaJ*	Chaperone protein dnaJ	TGGACCTGGGGATTATGGTA/CATGCCAACTGAAGCAAAGA	98	95.9	0.999
*TUBα*	Tubulin alpha	CGGAGACTTTTGTGCAGTGA/TCCTGAATGTCGTGCTTGAG	118	87.4	0.994
*TUBβ*	Tubulin beta	TGCAGACGAGGATGCTTATG/GCAATTGCAGAAGCACAGAA	97	106.0	0.998

**Table 2:** Expression stability of top four stable reference genes in RC15 evaluated by BestKeeper.

Gene	Geometric mean (Ct)	Arithmetic mean (Ct)	Minimum (Ct)	Maximum (Ct)	Coefficient of Variance ± Standard deviation (CV ± SD)	Pearson correlation coefficient (r )
*IDH*	25.47	25.59	23.25	32.02	7.87 ± 2.01	0.990
*RCA*	26.28	26.52	22.81	34.19	10.98 ± 2.91	0.985
*UBC2*	22.86	23.08	20.02	31.52	10.96 ± 2.53	0.972
*RPL2*	20.18	20.43	17.68	29.27	12.89 ± 2.63	0.964

**Table 3:** Expression stability of top four stable reference genes in RC30 evaluated by BestKeeper.

Gene	Geometric mean (C_t_)	Arithmetic mean (C_t_)	Minimum (C_t_)	Maximum (C_t_)	Coefficient of Variance ± Standard deviation(CV ± SD)	Pearson correlation coefficient (r )
*18S*	27.24	27.30	25.25	32.33	5.56 ± 1.52	0.989
*H2B*	23.28	23.36	21.23	28.19	6.74 ± 1.57	0.987
*IDH*	24.75	24.78	23.31	28.27	4.12 ± 1.02	0.985
*GAPDH*	24.30	24.35	22.63	28.23	5.18 ± 1.26	0.984

**Figure 1:**
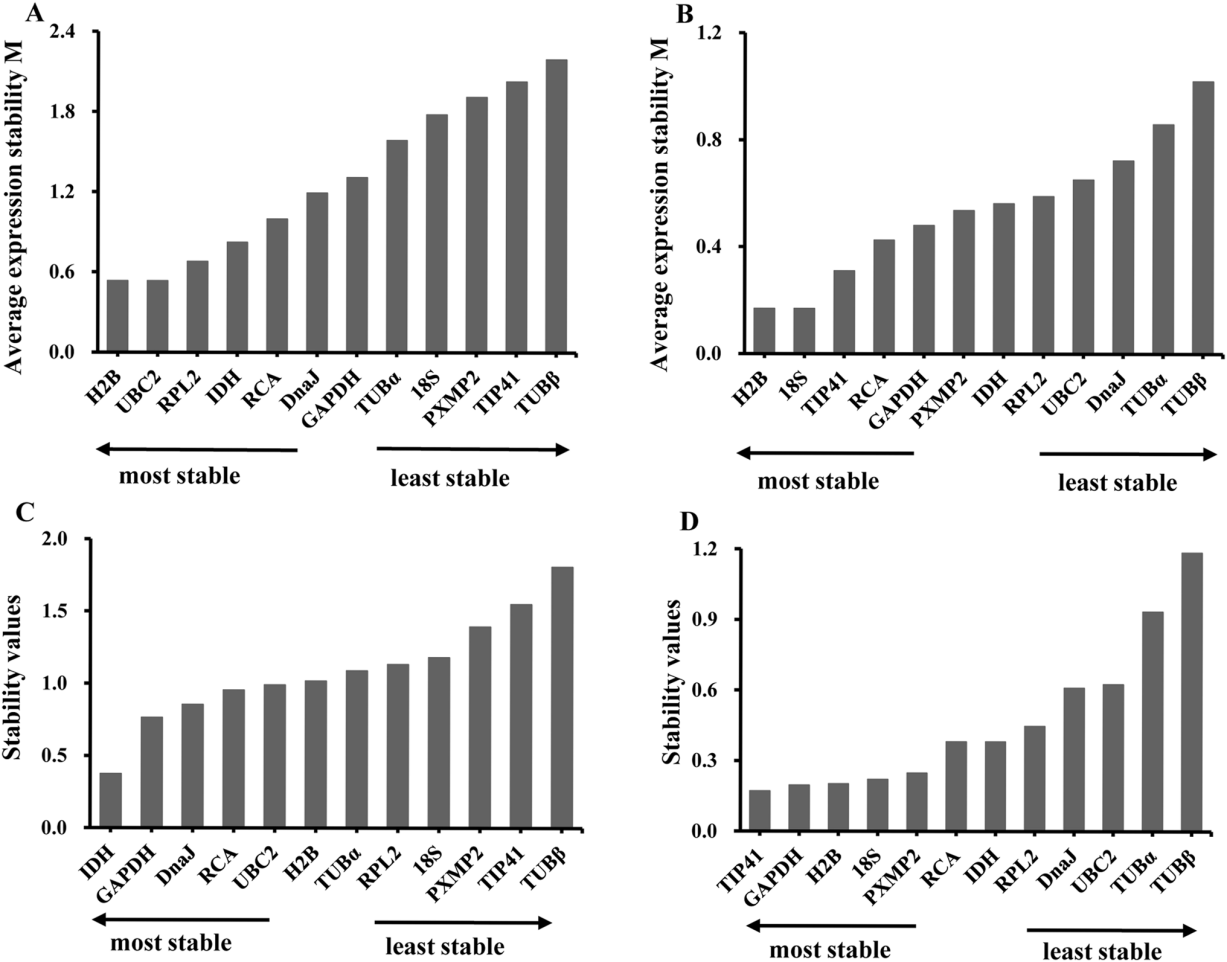
Ranking and expression stability values of the reference genes in RC15 and RC30 tissues by geNorm and NormFinder. (**A**) Reference genes in RC15 evaluated by geNorm. (**B**) Reference genes in RC30 evaluated by geNorm. (**C**) Reference genes in RC15 evaluated by NormFinder. (**D**) Reference genes in RC30 evaluated by NormFinder.

In the GeNorm analysis, *H2B* and *UBC2* had the lowest M values and thus the highest expression stability, followed by *Rpl2* and *IDH* in RC15 tissues (Fig. 1A). In GeNorm analysis of the RC30 tissues, *H2B* and *18S* were the most stable genes, followed by *TIP41* and *RCA* (Fig. 1B). The NormFinder analysis ranked *IDH*, *GAPDH*, *DnaJ* and *RCA* in the top positions for RC15 samples (Fig. 1C)*,* and in NormFinder analysis of the RC30 tissues, *TIP41* exhibited the most stable expression, followed by *GAPDH*, *H2B* and *18S* (Fig. 1D).

### Comprehensive ranking of the reference genes

The ranking order of each reference gene was obtained from 1 (most stable) to 11 (least stable) by geNorm and from 1 (most stable) to 12 (least stable) by BestKeeper and NormFinder. Different ranking of candidate genes among algorithms were revealed in other woody plants^22–24^. According to the arithmetic mean value of the ranking order of each gene, the top 4 stable genes were obtained. *IDH*, *UBC*, *RCA*, and *H2B* exhibited higher stability of gene expression in XRC15, and the mean rank value of the genes were 1.7, 3.0, 3.3, and 4.0 respectively (Table 4). *H2B* (2.0), *18S* (2.0), *TIP41* (2.7) and *GAPDH (3.3)* were the top 4 stable genes in XRC30 (Table 5).

**Table 4:** Comprehensive ranking of 12 reference genes in RC15.

Ranking order	GeNorm	NormFinder	BestKeeper	Comprehensive ranking (mean rank value)
1	*H2B/UBC2*	*IDH*	*IDH*	*IDH* (1.7)
2	*RPL2*	*GAPDH*	*RCA*	*UBC2* (3.0)
3	*IDH*	*DnaJ*	*UBC2*	*RCA* (3.3)
4	*RCA*	*RCA*	*RPL2*	*H2B* (4.0)

**Table 5:** Comprehensive ranking of 12 reference genes in RC30.

Ranking order	GeNorm	NormFinder	BestKeeper	Comprehensive ranking (mean rank value)
1	*H2B/18S*	*TIP41*	*18S*	*H2B* (2.0)
2	*TIP41*	*GAPDH*	*H2B*	*18S* (2.0)
3	*RCA*	*H2B*	*IDH*	*TIP41* (2.7)
4	*GAPDH*	*18S*	*GAPDH*	*GAPDH* (3.3)

### Phylogenetic analysis and functional prediction of ClMYB transcripts

**Figure 2:**
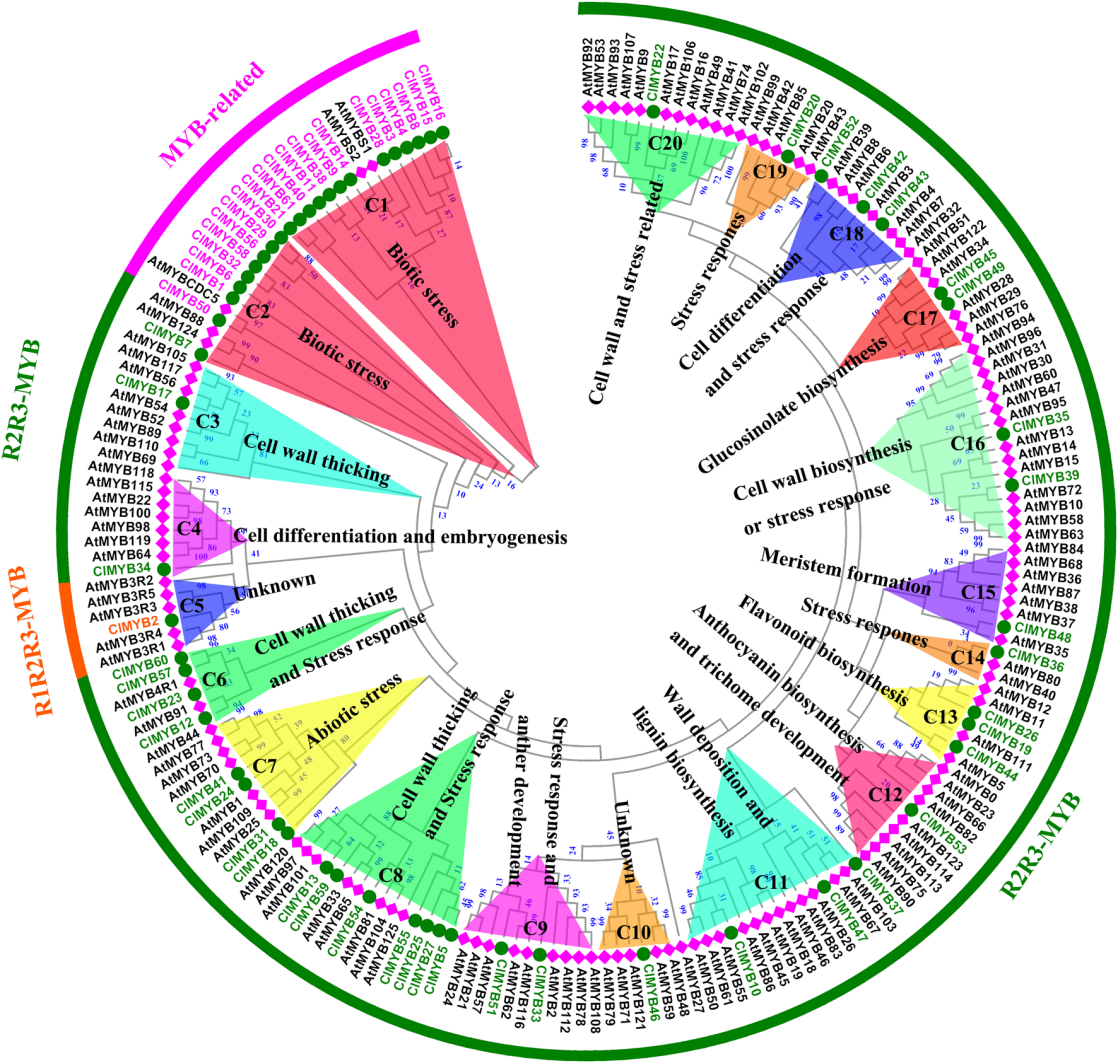
Evolutionary relationships and putative functions of the MYB proteins in *Cunninghamia lanceolata* (starting as Cl) based on the phylogenetic tree with MYB proteins in *Arabidopsis thaliana* (At). The circular unrooted tree was generated by Neighbor-Joining method in MEGA 7.0 with 1,000 bootstrap replicates, Jones-Taylor-Thornton (JTT) model and pairwise deletion treatment. The analysis involved all domain sequences of respective R2R3-MYB protein with 61 ClMYBs and 135 AtMYBs.

To evaluate the evolutionary relationships of the *ClMYB* transcripts, an NJ unrooted phylogenetic tree was constructed using amino acid sequences of 61 ClMYBs and 135 AtMYBs (Arabidopsis thaliana) (Fig. 2). Based on the number of MYB DNA-binding domain and phylogenetic relationships, we identified 21MYB-related proteins (1R-MYB), 39 R2R3-MYB proteins (2R-MYB), 1R1R2R3-MYB proteins (3R-MYB). According to the alignment result, all MYB members from the two species were subdivided into twenty subgroups (designated as C1-C20). All subgroups were common to the two species. 59 ClMYBs belonging to 18 function-annotated subgroups, and 2 ClMYBs belonging to two function-unknown subgroups were found. Because these *ClMYB* transcripts were captured from different woody tissues in RC of Chinese fir stem, *ClMYB* transcripts were involved in divergent functions including stresses response, cell differentiation, lignin biosynthesis, and secondary cell wall formation during heartwood formation of Chinese fir.

R2R3-MYB family genes have been conducted in depth studies and the function of genes are relatively clear^25–28^. To explore the phylogenetic relationships of the R2R3-MYB transcripts, two unrooted phylogenetic trees were constructed with 41 R2R3-MYBs (Chinese fir), 135 R2R3-MYBs (*Arabidopsis thaliana*), 194 R2R3-MYBs (*Populus trichocarpa*), and 142 R2R3-MYBs (*Eucalyptus grandis*) by NJ and ML method. A Similar topology was obtained between NJ tree (Fig. S4) and ML tree (Fig. S5). Some homologs between Chinese fir and *Arabidopsis thaliana* were clustered within the same subgroup may share similar functions. For example, *ClMYB* transcripts and *AtMYB* genes of the C7 subgroup in Fig. 2 (*ClMYB18*, *ClMYB24*, *ClMYB31*, *ClMYB41*, *AtMYB44*, and *AtMYB73*) and C8 subgroup (*ClMYB13*, *ClMYB54*, *ClMYB59*, *AtMYB33*, *AtMYB65*, and *AtMYB101*) were clustered within the same subgroup by different methods.

### *ClMYBs* expression profile in RC30 tissues

**Figure 3:**
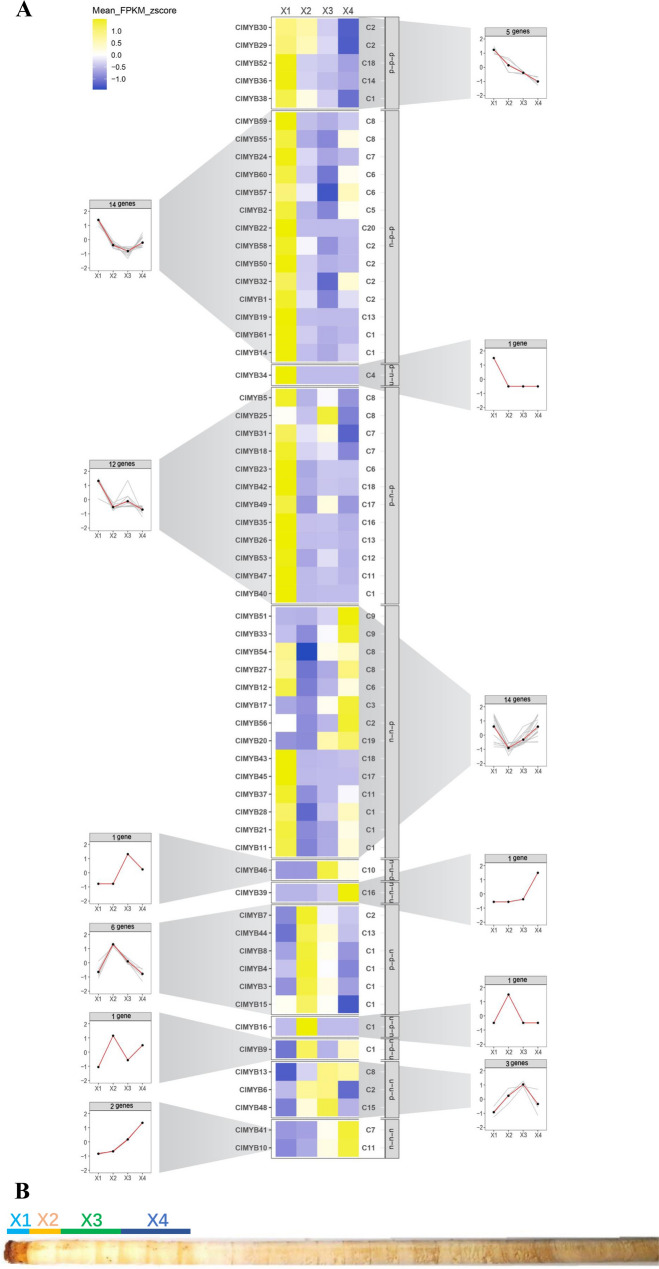
Heatmap of the RNAseq transcript abundance pattern of the 61 MYB transcripts from *Cunninghamia lanceolata* in four woody tissues clustered in 12 expression groups by *K-means*. (**A**) Heatmap of the RNAseq transcript abundance pattern. Transcript name is included to the left of the heatmap and the short name of the phylogenetic subgroup is shown to the right. *K-means* clusters were performed based on fragments per kilobase of exon per million fragments mapped (FPKM) values adjusted through z-score standardization. (**B**) Localization of different woody tissues used for RNA-seq and qRT-PCR experiment of 30 years old Chinese fir stem. X1, cambium zone; X2, outer sapwood; X3, inner sapwood; X4, transition zone.

**Figure 4:**
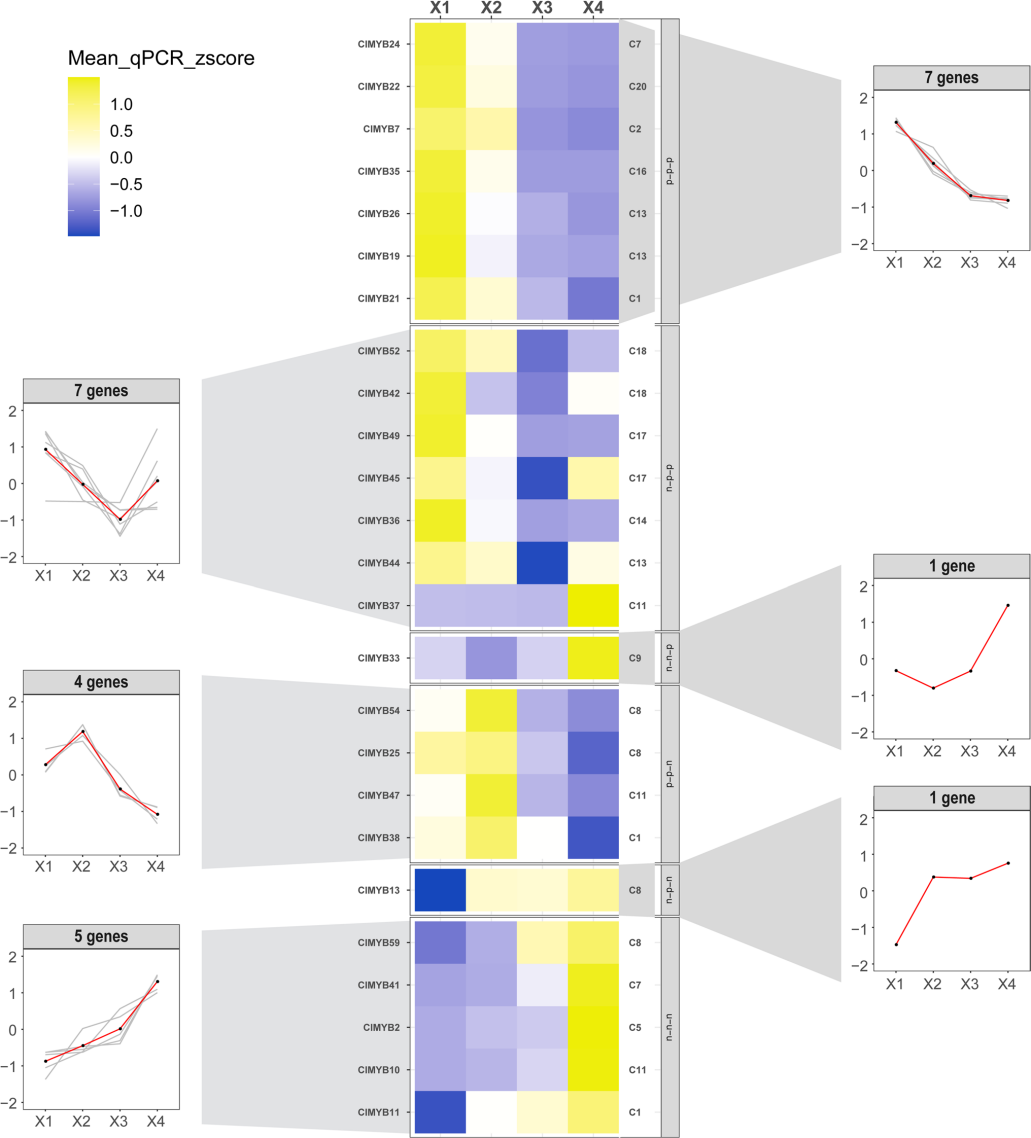
The heatmap of the transcript abundance patterns validated by qRT-PCR of the 25 *MYB* transcripts from *Cunninghamia lanceolata* in four woody tissues. Twenty-five *ClMYB* transcripts were clustered in 6 expression groups by *K-means*. Data was analyzed by the 2 ^−ΔΔCt^ method and adjusted through z-score standardization. Transcript name is included to the left of the heatmap and the short name of the phylogenetic subgroup is shown to the right.

*ClMYB* transcript abundance was analysed by RNA-seq in 4 woody tissues of Chinese fir (Fig. 3). Based on RNA-seq expression, 61 *ClMYB* transcripts revealed 12 expression clusters by *K-means*. Thirty-five *ClMYB* transcripts had different expression patterns (fold change > 2, *P* < 0.05) in woody tissues (Table S1). Expression of 25 *ClMYB* transcripts validated by qRT-PCR in the same woody tissues as RNA-seq. Normalized to *H2B*, *18S*, *TIP41*, and *GAPDH*, a heatmap was constructed with the expression level of *ClMYB* transcripts (Fig. 4). Based on qPCR-based expression, 25 *ClMYB* transcripts revealed six expression clusters by *K-means*.

Three *ClMYB* transcripts (*ClMYB10*, *ClMYB33*, and *ClMYB41*) had the same expression pattern with the *K-means* cluster by both qRT-PCR and RNA-seq. Based on six pairwise comparisons in the four woody tissues (X1, X2, X3, and X4), a total of 18 differentially expressed transcripts were identified by RNA-seq (Table S1). Eight differentially expressed transcripts identified by qRT-PCR were also identified by RNA-seq. *ClMYB22* was the transcript with the greatest fold change based on RNA-seq data and it was up-regulated with a 6525-fold change in X1 compared to X2. *ClMYB22* was also the transcript with the greatest fold change based on qRT-PCR data, but the greatest fold change was up-regulated with an 8714-fold in X1 compared to X4. Nine *ClMYB* transcripts (*ClMYB19*, *ClMYB21*, *ClMYB22*, *ClMYB24*, *ClMYB26*, *ClMYB35*, *ClMYB36*, *ClMYB42*, *ClMYB45*, *ClMYB49*, and *ClMYB52*) had same preferential expression detected by two methods. These transcripts all highly expressed in X1, which indicate the results of transcript abundance detected by qRT-PCR were not highly consistent with the RNA-seq in specific xylem tissues (X2, X3, and X4) representing the heartwood formation. Because qRT-PCR and RNA-Seq were conducted with the RNA taken from different increment cores, it reduced the consistency of gene expression patterns detected by the two methods.

## Discussion

In molecular biological research, transcript expression analysis has been an effective basic strategy to understand the establishment of cellular states and predict gene function^29^. qRT-PCR is a commonly used method to quantify gene expression with appropriate reference genes as internal controls and it is unreliable to apply a universal reference gene for different tissues and treatments^30^. HW formation is described as a form of PCD^4^. Expression of genes involved in secondary metabolite biosynthesis and PCD change greatly from SW and TZ toward HW regions^24^. At present, little attention has been paid to selecting stable and appropriate reference genes for the gene expression normalization in RC of gymnosperm.

We determined the expression stability of 12 reference genes in RC of Chinese fir stem by three statistical algorithms. Pearson coefficient of correlation (r) was used as the main reference value for gene stability in Bestkeeper. GeNorm recommends selecting genes with an M value < 1.5. NormFinder provides a stability value of each reference gene by estimating both intra- and inter- group expression variation^12,32,33^. According to the three algorithms, these results show that the expression of the candidate genes is more variable in RC15 than in RC30. The accuracy of target gene expression level may be influenced by using just a single reference gene in qRT-PCR^7^. Integrated ranking of reference genes stability from the three software and utilizing multiple reference genes for the normalization is necessary and shows up as a more reliable accuracy of target gene expression level in qRT-PCR^34^. Compared to the calculation of geometric mean, arithmetical mean of ranking values by the different algorithms is a more comprehensive way to create a consensus ranking^35^.

In our study, *IDH*, *UBC2*, *RCA*, and *H2B* were the most stable reference genes in RC15. *H2B*, *18S*, *TIP41*, and *GAPDH* were the most stable reference genes in RC30. *H2B* was considered as the stable reference genes from adventitious rooting in *Eucalyptus globulusl*^36^. *IDH* was used as reference genes in gene expression experiments to analyze flowers of *Pinus massoniana* L. in different developmental phases^37^. *GAPDH* was the suitable reference gene for xylem tissues in *Camellia oleifera*^38^. *18S* was one of stable reference genes in different tissues of *Taxus spp*^39^. *UBC2* was identify the best reference genes for *Osmanthus fragrans*^40^. *RCA* was upregulated in response to salt stress for cucumber^41^. *TIP41* was the stable reference gene for low-temperature stress in *Populus trichocarp*^42^.

Vascular plants developed specialized conductive tissues in xylem vessels characterized by SCW deposition to increase water conductive activity^43^. MYBs play critical roles in controlling xylem cell differentiation and SCW biosynthesis pathway^44^. Homology classification is important to predict the gene function and genes in the same subgroup based on the sequence are thought to have relatively similar roles^45^. Three MYB subgroups (S5, S6 and SAtM5) are identified by phylogenetic approach to belonging to putative woody-expanded subgroups from the woody perennial species. These woody-expanded subgroups are involved in the phenylpropanoid pathway^27^. *ClMYB26* and *ClMYB49* were identify to belong to S5. *ClMYB19* and *ClMYB36* belong to *SAtM5*. These four MYB transcripts from Chinese fir were preferentially expressed in the cambium-enriched region of Chinese fir and may be involved in regulating of the phenylpropanoid pathway.

*ClMYB* transcript and *AtMYB* genes of the C3 subgroup (ClMYB17, AtMYB56, AtMYB105, and AtMYB117) were clustered into the same subgroup with different methods. These *MYB* genes of Arabidopsis are involved in the cell wall metabolism^46,47^ and organ patterning^48,49^. *ClMYB17* may have similar functions and was preferentially expressed in the transition zone of Chinese fir. Some *ClMYB* transcripts and *AtMYB* genes of C8 subgroup (*ClMYB13*, *ClMYB54*, *ClMYB59*, *AtMYB33*, *AtMYB65*, and *AtMYB101*) were clustered into the same subgroup. These MYB genes of Arabidopsis are involved in the programmed cell death process^50^. Although *ClMYB13*, *ClMYB54*, and *ClMYB59* maybe involved in programmed cell death process, the transcripts with different expression patterns in radial change of Chinese fir stem have different functions.

Some *ClMYB* transcripts and *AtMYB* genes of C2 subgroup (*ClMYB7*, *AtMYB88*, and *AtMYB124*), C7 subgroup (*ClMYB18*, *ClMYB24*, *ClMYB31*, *ClMYB41*, *AtMYB44*, and *AtMYB73*), C9 subgroup (*ClMYB33*, *ClMYB51*, *AtMYB62*, and *AtMYB108*), C16 subgroup (*ClMYB39*, *AtMYB13*, *AtMYB14*, and *AtMYB15*), C18 subgroup (*ClMYB42*, *ClMYB43*, *ClMYB52* and *AtMYB4*), C19 subgroup (*ClMYB20* and *AtMYB20*) were clustered into each subgroup. *AtMYB* genes of these subgroups involve in transducing or conferring resistance to abiotic stresses^51–56^. *ClMYB* transcripts of these subgroups may have similar functions. *ClMYB18*, *ClMYB20*, *ClMYB24*, *ClMYB31*, *ClMYB52*, *ClMYB42*, and *ClMYB43* were preferentially expressed in the cambium zone of Chinese fir. *ClMYB7* was highly expressed in outer sapwood. *ClMYB33*, *ClMYB39*, *ClMYB41*, and *ClMYB51* showed higher expression in the transition zone. The different expression pattern of these *ClMYBs* indicates the transcripts involved in stress responses at the different stages during heartwood formation of Chinese fir.

## Methods

### Collection of plant material

Samples excised at breast height (1.3 m) of 26 years old Chinese fir stem were successfully used to study cellular changes from cambium to heartwood in Chinese fir^57^. Increment cores were taken from 35 years old Taiwanina (*Taiwania cryptomerioides* Hayata) stems to analyze the expression profiling of the heartwood formation process^31^. In this study, increment cores were sampled from breast height of three well-grown individuals (of each) of 15 years old and 30 years old Chinese fir stems in summer (August 2019). The radial change tissues of outer sapwood, inner sapwood, and the transition zone were taken from 15 years old Chinese fir stems. The tissues of outer sapwood, middle sapwood, inner sapwood, and the transition zone were taken from 30 years old Chinese fir stems. While these above samples were collected, all samples were immediately frozen in liquid nitrogen and later stored in − 80 °C for subsequent experiments. Increment cores were taken from our test plantation in Rongshui county of China (25°04′48.6″ N, 108°15′16.3″ E). Since the collected materials were taken from our (own) test plantation, there will be no permission needed. The study complies with relevant institutional, national, and international guidelines and legislation.

### Total RNA extraction and cDNA synthesis

Total RNA from different samples was isolated by RNAprep Pure Plant Kit (Tiangen, China) without DNA contamination following the manufacturer’s protocol. RNA integrity was checked on 1% agarose gel electrophoresis. The quantity and quality of total RNA were measured by a NanoDrop 2000 Spectrophotometer (Thermo Scientific, US). RNA was homogenized with RNase-free water. Total RNA (500 ng per samples) was reversely transcribed to first-strand of cDNA by PrimeScript RT reagent Kit (TaKaRa, Japan). All cDNA samples were stored at − 20 °C.

### qRT-PCR

The full-length transcriptome from equal amounts of mixed RNA obtained from 4 woody tissues (X1, X2, X3, and X4) of one 30 years old Chinese fir was sequenced on the PacBio Sequel platform from our laboratory (SRA accession: PRJNA760952). The consensus sequence was obtained. A total of 12 RNA-seq libraries, with 4 woody tissues (X1, X2, X3, and X4) per tree for three 30 years old individuals were sequenced by Illumina HiSeqX platform (SRA accession: PRJNA760952). According to the literature of reference genes selection^36,58,59^ and our previous RNA-Seq data of 4 woody tissues (Novogene, China), 12 candidate reference genes were screened based on rules of q-value ≥ 0.05, FPKM ≥ 5, and Fold Change < 2. Specificity of primer pair of 12 candidate reference genes and 25 *ClMYB* transcripts were controlled by performing melting curves of the qRT-PCR products (Tianlong, China). Melting curves showed a single peak indicates the absence of side products and primer dimers.

Nucleotide sequence data of 12 candidate reference genes and the primers of 25 *ClMYBs* were listed in Table S2. The conserved domains of their mRNA sequences were identified using the NCBI database and local blast of PacBio-Seq data, the primers for qRT-PCR amplification were then designed on the web using Primer 3.0 (http://www.primer3plus.com/primer3web/primer3web_input.htm) with the following criteria: the primers should be with limited lengths of 20–24 bp, melting temperatures (Tm) in the range of 55–62˚C, GC contents varying from 45 to 55%, and product lengths of 80–250 bp, other parameters were set as default. qRT-PCR assay was performed in a volume of 20 μL with three technical replicates on Gentier 32R real-time PCR system (Tianlong, China) using TB Green Premix Ex Taq (TaKaRa, Japan). The reaction was performed at the following conditions: initial denaturation at 95 °C for 30 s, followed by 45 cycles of 95 °C for 5 s, 55 °C for 10 s, and 72 °C for 10 s.

### Phylogenetic analysis of the ClMYB proteins

The full-length transcriptome of 4 woody tissues (X1, X2, X3, and X4) from one 30-year-old Chinese fir was obtained on the PacBio Sequel platform (SRA accession: PRJNA760952). To derive annotation information and identify the *MYB* transcripts, the unique isoforms were mapped to Swiss-Prot (https://www.uni-prot.org/uniprot/). The online software SMART (http://smart.embl.de/) were used to identify the domain sequences of all MYB proteins. The names and accession numbers of *MYBs* in the other three species were based on previous research^25–28^. According to accession numbers, the protein sequence of MYBs was available in *Arabidopsis thaliana* (Plant Transcription Factor Database: http://planttfdb.gao-lab.org/), *Populus trichocarpa* (PoplarGene: http://bioinformatics.caf.ac.cn/PoplarGene), and *Eucalyptus grandis* (NCBI Genbank: https://www.ncbi.nlm.nih.gov/genbank/). The online SMART software (http://smart.embl.de/) were used to identify the amino acid sequence of domains of each MYB protein. The protein sequence of 61 ClMYBs and the whole domain of each MYB protein are shown in Table S3.

Based on the whole domain sequence of each MYB protein, neighbor-joining (NJ) phylogenetic trees were generated by MEGA 7.0 with 1,000 bootstrap replicates, Jones-Taylor-Thornton (JTT) model and pairwise deletion treatment. Based on the whole domain sequence of each MYB protein, Maximum Likelihood (ML) phylogenetic tree was generated by MEGA 7.0 with 1,000 bootstrap replicates, Jones-Taylor-Thornton (JTT) model and complete deletion treatment. The biological functions of ClMYBs were predicted by homologous MYBs with validated function within the same phylogenetic tree clade.

### RNAseq and qRT-PCR expression analysis

The correlation coefficient (R^2^) and amplification efficiency (E) of primer pairs were counted by the linear regression model with a series of fivefold dilution of first-strand cDNA (0.5 μg/μL). The PCR efficiency was calculated by the equation E = (10^[-1/slope]^ − 1) × 100%. The expression stability of candidate reference genes was calculated using geNorm, NormFinder, and Bestkeeper^12,55,56^. We used geometric mean of 4 selected reference genes of Chinese fir for the normalization of data. The relative expression levels were evaluated by 2^−ΔΔCT^.

RNA-seq data from 4 different woody tissues (X1, X2, X3, and X4) per tree for three 30 years old individuals were sequenced by Illumina HiSeqX platform (SRA accession: PRJNA760952). Mean fragments per kilobase of exon per million fragments mapped (FPKM) values per transcript were calculated for each tissue. *K-means* clusters performed with Perl were based on FPKM values adjusted through z-score standardization. Six pairwise comparisons were performed to identify DEGs: (1) X1 versus X2; (2) X1 versus X3; (3) X1 versus X4; (4) X2 versus X3; (5) X2 versus X4; and (6) X3 versus X4. Differential expression analysis by RNA-seq and qRT-PCR were based on rules of fold change > 2 and *P* < 0.05. Analysis of z-score standardization and ANOVA and drawing of heatmap and trendline was performed by R package.

## Supplementary Information

Supplementary Information 1.

Supplementary Information 2.

Supplementary Information 3.

Supplementary Information 4.

Supplementary Information 5.

Supplementary Information 6.

Supplementary Information 7.

Supplementary Information 8.
