# Supplementary material for: Evaluation of reference genes and characterization of the MYBs in xylem radial change of Chinese fir stem
Source: Sci Rep. 2022 Jan 7;12:258. doi: 10.1038/s41598-021-04406-1 (PMC8741804; doi:10.1038/s41598-021-04406-1)
Supplement: Supplementary file 1 — Supplementary Information 1. [file 41598_2021_4406_MOESM1_ESM.pdf]

Evaluation of reference genes and characterization of the *MYBs* in xylem radial change of Chinese fir stem

Kui-Peng Li[1] · Wei Li[2] · Gui-Yun Tao[3] · Kai-Yong Huang[1]✉

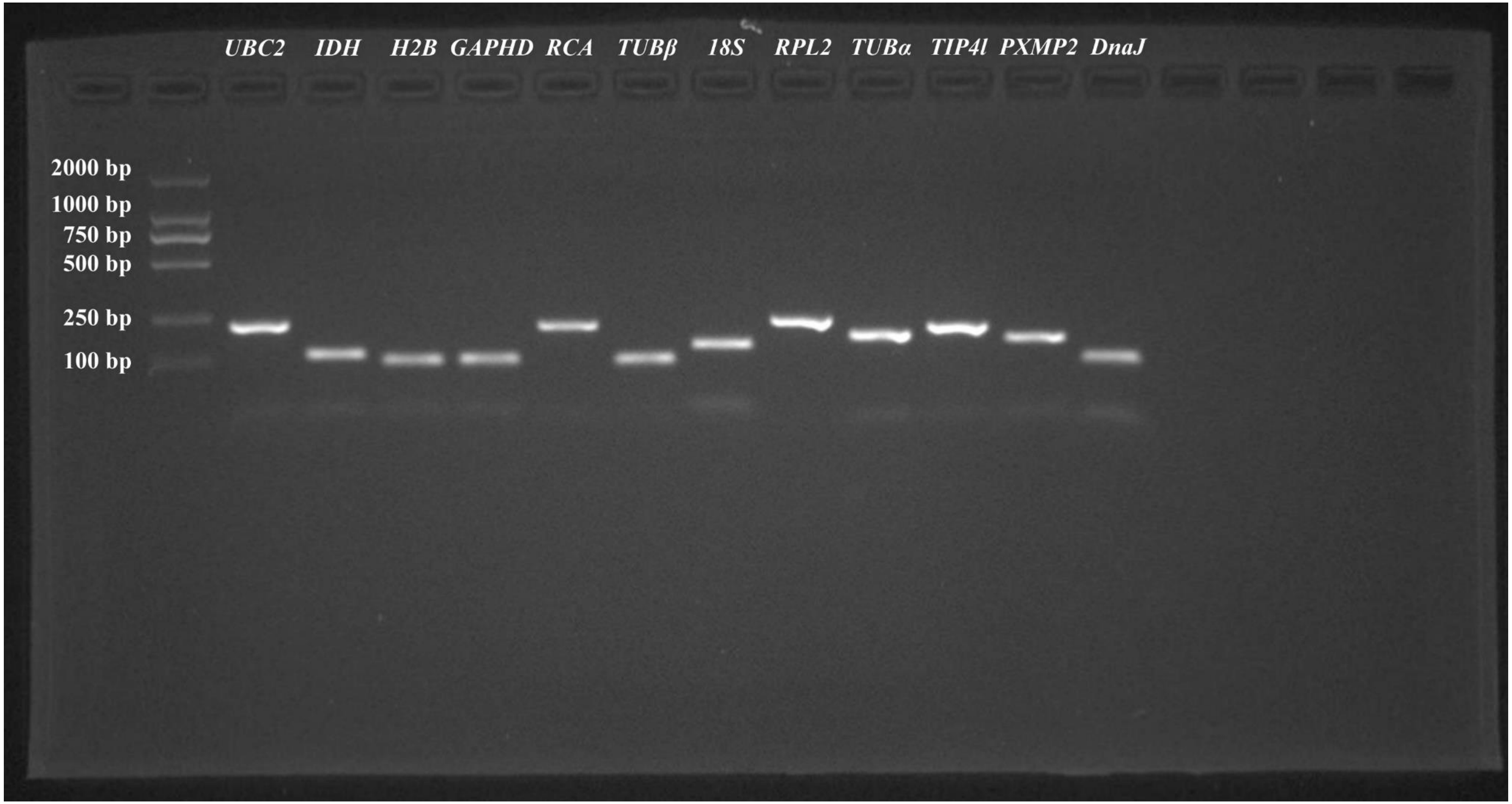

Supplementary figure S1. Primers validation by qPCR products of each target gene on a 2% agarose gel. The gel was detected with ChemiDoc MP Imaging System (BioRad). bp = base pairs.
