# Supplementary material for: Evaluation of reference genes and characterization of the MYBs in xylem radial change of Chinese fir stem
Source: Sci Rep. 2022 Jan 7;12:258. doi: 10.1038/s41598-021-04406-1 (PMC8741804; doi:10.1038/s41598-021-04406-1)
Supplement: Supplementary file 3 — Supplementary Information 3. [file 41598_2021_4406_MOESM3_ESM.pdf]

Evaluation of reference genes and characterization of the *MYBs* in xylem radial change of Chinese fir stem

Kui-Peng Li[1] · Wei Li[2] · Gui-Yun Tao[3] · Kai-Yong Huang[1*]

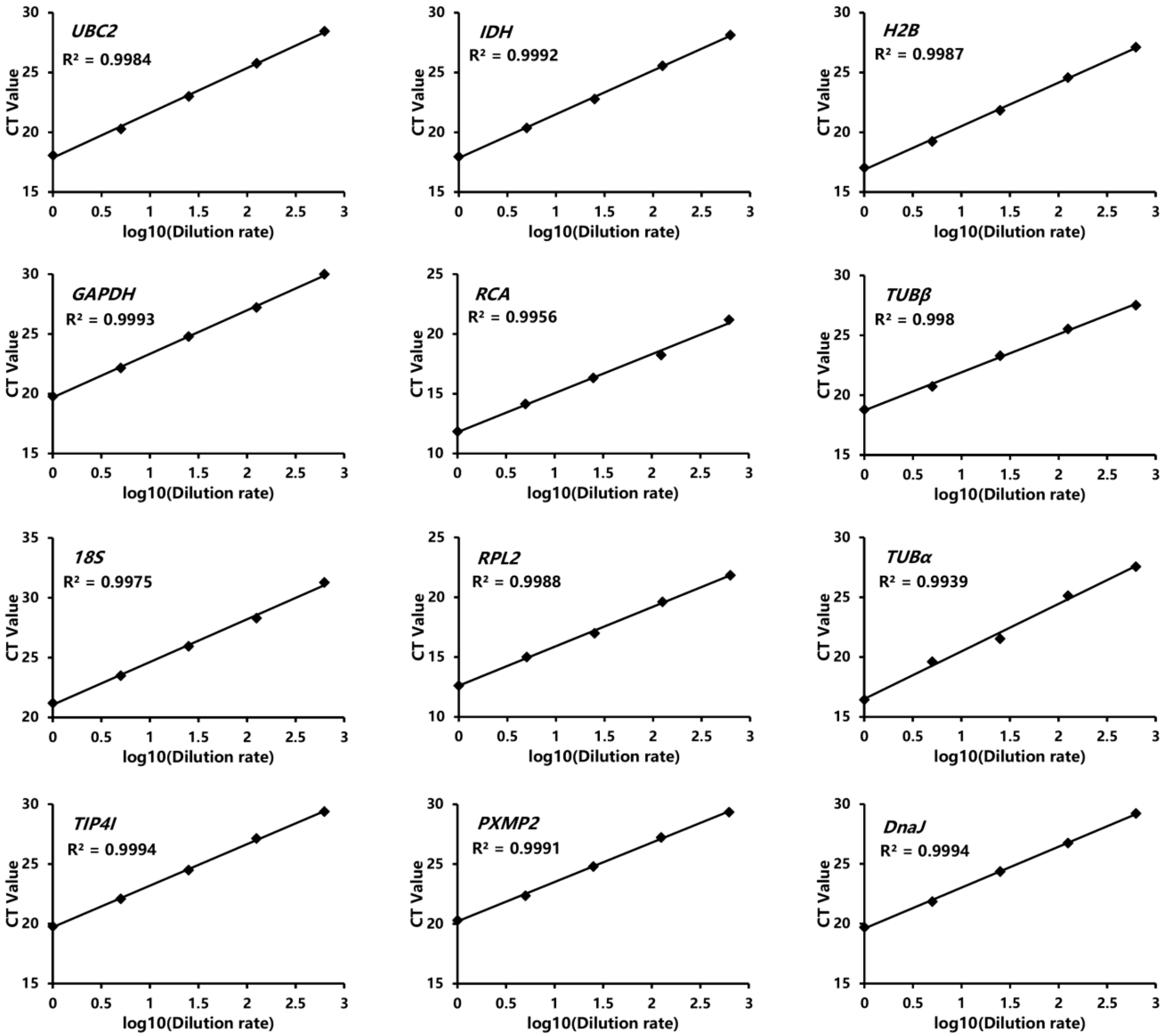

Supplementary figure S3. Correlation coefficient (R^2) of the selected reference genes. R^2 of primer pairs of reference genes were counted by the linear regression model with a series of 5-fold dilution of first-strand cDNA (0.5 μg/μL).
