# Supplementary material for: Evaluation of reference genes and characterization of the MYBs in xylem radial change of Chinese fir stem
Source: Sci Rep. 2022 Jan 7;12:258. doi: 10.1038/s41598-021-04406-1 (PMC8741804; doi:10.1038/s41598-021-04406-1)
Supplement: Supplementary file 5 — Supplementary Information 5. [file 41598_2021_4406_MOESM5_ESM.pdf]

Evaluation of reference genes and characterization of the *MYBs* in xylem radial change of Chinese fir stem

Kui-Peng Li[1] · Wei Li[2] · Gui-Yun Tao[3] · Kai-Yong Huang[1]✉

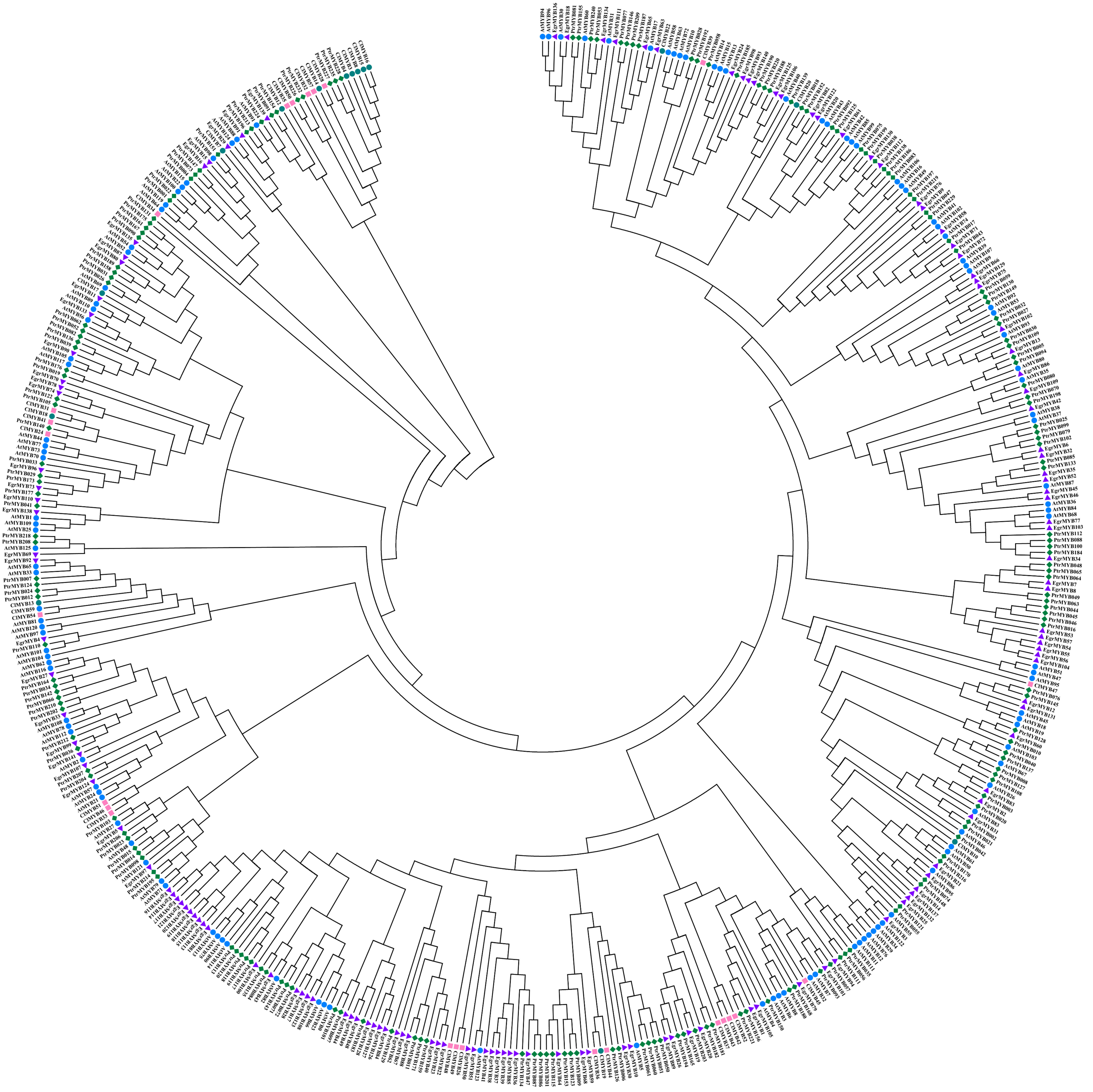

Supplementary figure S5. Maximum Likelihood phylogenetic tree constructed using predicted amino acid sequences of R2R3-MYB proteins. Phylogenetic trees were constructed with all domain sequence of respective 41 R2R3-MYBs in *Cunninghamia lanceolata* (starting as Cl)), 135 R2R3-MYBs in *Arabidopsis thaliana* (At), 194 R2R3-MYBs in *Populus trichocarpa* (Pt), and 142 R2R3-MYBs in *Eucalyptus grandis* (Eg). Phylogenetic tree was generated by Maximum Likelihood method in MEGA 7.0 with Jones-Taylor-Thornton (JTT) model and complete deletion treatment.
